# Author Correction: A systems biology-based investigation into the therapeutic effects of Gansui Banxia Tang on reversing the imbalanced network of hepatocellular carcinoma

**DOI:** 10.1038/s41598-022-22833-6

**Published:** 2022-11-04

**Authors:** Yanqiong Zhang, Xiaodong Guo, Danhua Wang, Ruisheng Li, Xiaojuan Li, Ying Xu, Zhenli Liu, Zhiqian Song, Ya Lin, Zhiyan Li, Na Lin

**Affiliations:** 1 Institute of Chinese Materia Medica, China Academy of Chinese Medical Sciences, Beijing, 100700 China; 2 302 Hospital of PLA, Beijing, 100039 China; 3 Institute of Basic Theory, China Academy of Chinese Medical Sciences, Beijing, 100700 China

Correction to: *Scientific Reports* 10.1038/srep04154, published online 24 February 2014

This Article contains errors.

As a result of figure assembly errors, in Figure 5A and Supplementary Figure 4F the images used to represent the treatment condition were incorrectly derived from the control dataset. The correct Figure 5 is shown below as Figure 1.

**Figure 1:**
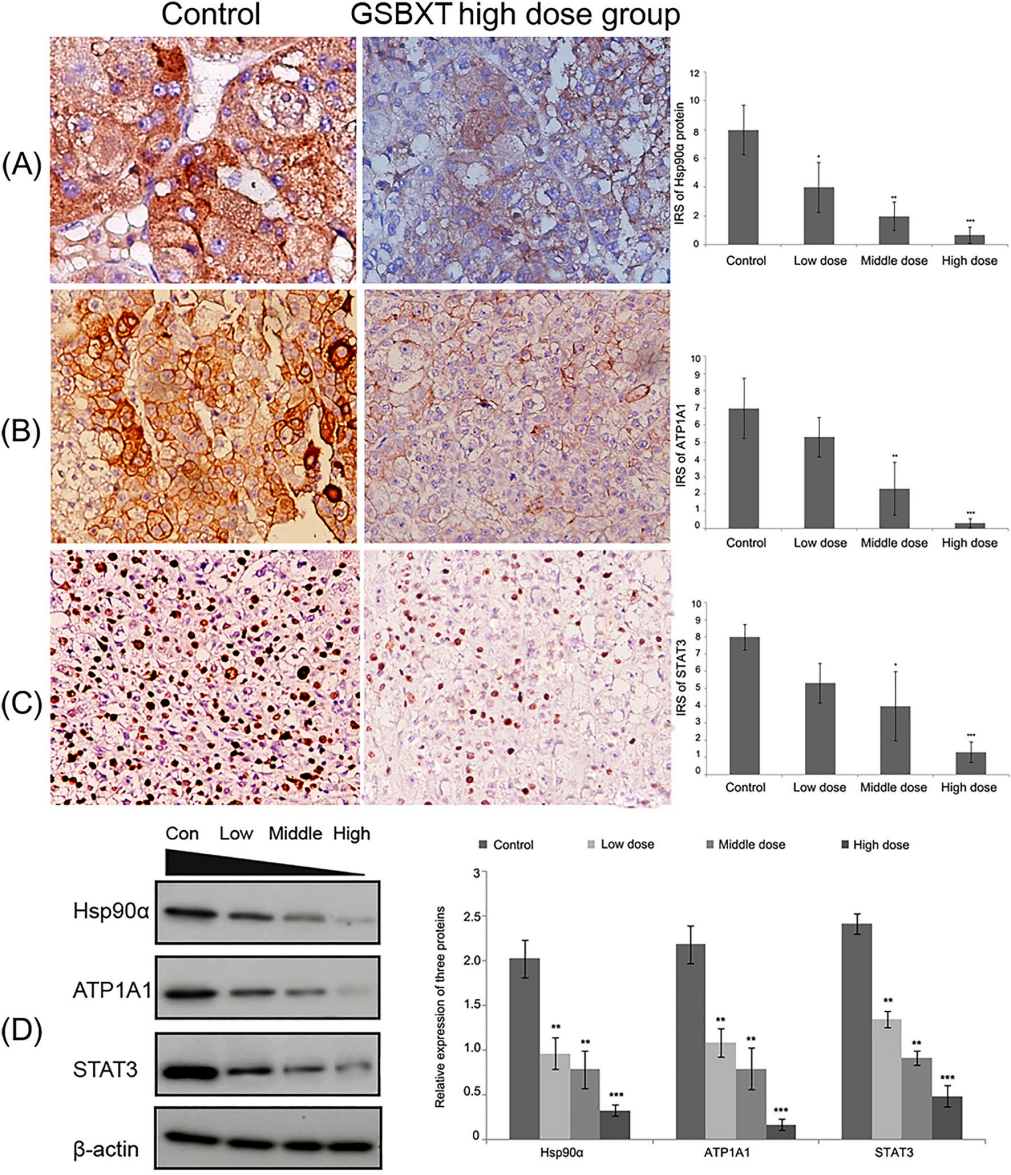
The corrected version of Figure 5.

The corrected Figure S4 is shown below as Figure 2.

**Figure 2:**
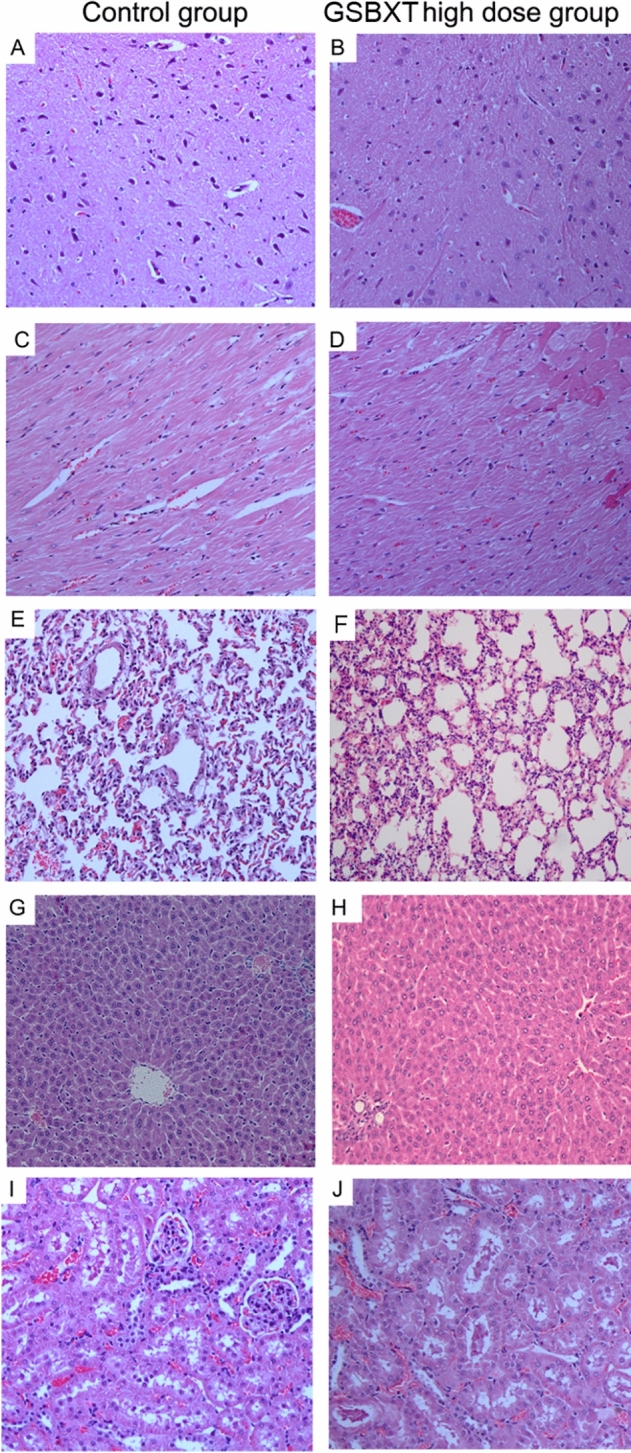
The corrected version of Figure S4

